# Corona-Krise fordert Wohnungspolitik heraus

**DOI:** 10.1007/s10273-020-2699-y

**Published:** 2020-07-18

**Authors:** Holger Cischinsky, Philipp Deschermeier, Max-Christopher Krapp, Martin Vaché

**Affiliations:** Institut Wohnen und Umwelt GmbH (IWU), Rheinstraße 65, 64295 Darmstadt, Deutschland

**Keywords:** R21, 31, H55

## Abstract

Die Corona-Pandemie führt auch im Bereich des Wohnens zu gravierenden Effekten. Kurzfristig hat die Bundesregierung mit temporären Ausnahmeregelungen bei den subjektorientierten Instrumenten sowie im Miet- und Darlehensrecht reagiert. Es zeigt sich jedoch, dass weitere temporäre Regelungen geboten sind. Aufgrund der großen Relevanz von nachfrageseitigen Entwicklungen ist im Verlauf der Rezession mit ausgeprägten Preisrückgängen auf den Wohnungsmärkten zu rechnen, die potenziell jedoch durch eine langfristig neu einsetzende Arbeitsmigration revidiert werden. Die Wohnungspolitik ist daher gut beraten, ihre Wohnungsbauaktivitäten zu stabilisieren bzw. antizyklisch auszurichten, um die langfristigen Wohnungsbedarfe auch bei wieder auflebender Zuwanderung decken zu können.

## Abstract (en)

The coronavirus pandemic and the associated containment measures are likely to have serious effects on housing. In the short term, the German Federal Government has reacted with temporary exemptions for subject-oriented instruments as well as for rental and lending regulations. However, it has become apparent that further temporary regulations are needed within the scope of social security of housing. In the course of the recession, pronounced price declines on the housing markets are to be expected due to the great relevance of demandside developments. However, longterm labour migration will potentially revise the price declines. Housing policymakers would therefore be advised to stabilise housing construction activities or to orient them countercyclically in order to be able to cover longterm housing needs that will emerge by resurgent immigration.

Die Maßnahmen zur Eindämmung der durch das Coronavirus SARS-CoV-2 ausgelösten Pandemie beeinträchtigen das gesamte Wirtschaftsleben und es kann als gesichert angesehen werden, dass die Corona-Krise eine weltweite Rezession auslösen wird. Für Deutschland rechnet die Bundesregierung für das laufende Jahr mit einem (preisbereinigten) Rückgang des Bruttoinlandsprodukts (BIP) um 6,3 % (BMWi, 2020). Und ob der für 2021 erwartete BIP-Zuwachs in Höhe von 5,2 % eintritt, bleibt abzuwarten.

Eine Rezession dieses Ausmaßes wirkt sich auch auf Wohnungsmärkte aus. Aufgrund von Einnahmeausfällen stehen bereits kurzfristig viele Mieter vor dem Problem, ihre Miete nicht zahlen zu können, während Immobilieneigentümer, insbesondere selbstnutzende Eigenheimbesitzer, in finanzielle Bedrängnis geraten können, wenn Hypothekenkredite zu bedienen sind. Und rezessionsbedingt ist spätestens mittel- bis langfristig mit Effekten auf der Angebots-, vor allem aber auf der Nachfrageseite zu rechnen, die aufgrund der Trägheit von Wohnungsmärkten zwar erst zeitverzögert eintreten, dafür aber mutmaßlich noch Jahre nach dem Ende der Pandemie andauern werden.

**Dr. Holger Cischinsky**, **Dr. Philipp Deschermeier**, **Dr. Max-Christopher Krapp** und **Martin Vaché**, Dipl.-Ing. M.Sc., sind wissenschaftliche Mitarbeiter beim Institut Wohnen und Umwelt in Darmstadt.

Mit Sofortmaßnahmen versucht die Bundesregierung, unmittelbar eintretende soziale Schieflagen abzufedern. Die zeitlich befristeten Maßnahmen setzen im Bereich des Wohnens am Miet- und Darlehensrecht sowie am Sozialrecht, vor allem an der Absicherung angemessenen Wohnens in der Grundsicherung, an. Rezessionsbedingte Auswirkungen auf die Wohnungsmärkte werden allerdings erst nach dem Ablauf dieser Fristen spürbar sein.

## Kurzfristige Marktentwicklungen

Auf kurze Sicht führen die Corona-bedingten Kontaktsperren durch Kurzarbeit, Arbeitslosigkeit oder eingeschränkte Geschäftstätigkeit bei vielen Haushalten zu Einkommensverlusten. Wohnen betroffene Haushalte zur Miete, ist ihre Mietzahlungsfähigkeit gefährdet, leben sie im Eigenheim, fehlen unter Umständen Mittel zur Bedienung von Hypothekendarlehen. Im Extremfall können starke Einkommensrückgänge, und seien sie auch nur temporär, zum Verlust der Mietwohnung oder des nicht abgezahlten Eigenheims führen. Hier setzen die wohnungspolitischen Sofortmaßnahmen an. Auch durch den Rückgang des Vermietungs- und Verkaufsgeschehens infolge von Kontaktverboten sind die Wohnungsmärkte unmittelbar von der krisenhaften Entwicklung betroffen. Die Auswirkungen auf die Miet- und Kaufpreise abzuschätzen, ist jedoch schwierig, da zu wenige Preisinformationen verfügbar sind. So dürften erschwerte Bedingungen bei Mieterhaushalten, die auf einen Wohnungswechsel angewiesen sind, einerseits eine höhere Bereitschaft auslösen, sich schneller für ein weniger günstiges Angebot zu entscheiden. Andererseits wird das Interesse der Vermieter, verlängerte Vermarktungszeiträume aufgrund geringerer Besichtigungsintensität zu vermeiden, vermutlich eher zu Preiszugeständnissen führen.

### Mietrecht

Im Bereich des Mietrechts schränken die Sofortmaßnahmen das Kündigungsrecht der Vermieterseite ein (BGBl, 2020a). Demnach dürfen Vermieter einen Mietvertrag nicht kündigen, weil Mieter im Zeitraum vom 1. April 2020 bis 30. Juni 2020 trotz Fälligkeit die Miete nicht leisten, wenn die Mieter dies mit den Auswirkungen der Corona-Krise begründen. Mietschulden aus dem erwähnten Zeitraum müssen bis zum 30. Juni 2022 beglichen werden, sonst kann den Mietern gekündigt werden.

Auch wenn diese Stundungsmöglichkeit als Maßnahme ausgewogen erscheint, ist zu berücksichtigen, dass Stundungen Privatvermieter sensibel treffen können. Diesen gehören knapp zwei Drittel der vermieteten Wohnungen in Deutschland und meist verfügen sie nur über geringe Bestandsumfänge. So kam eine repräsentative Befragung unter Privateigentümern von Mehrfamilienhausmietwohnungen zum Ergebnis, dass rund 57 % dieser knapp 5 Mio. Personen nur über eine einzige Mietwohnung verfügen (Cischinsky et al., 2015). Eine Risikostreuung ist bei so geringen Beständen nicht möglich, wodurch Mietstundungen zum (temporären) Wegbrechen der gesamten Mieteinnahmen führen können. Ferner ist der Anteil der Selbständigen unter den Privatvermietern höher als in der Gesamtbevölkerung, sodass ausbleibende Mietzahlungen insbesondere einen Bevölkerungsteil treffen, der auch schon in anderer Hinsicht durch die Corona-Krise stark belastet wird. Eine etwaige Verlängerung des Stundungszeitraums und der Rückzahlungsfrist wäre daher genau abzuwägen.

### Darlehensrecht

Durch die Niedrigzinspolitik im Euroraum wurde der Erwerb eines Eigenheims für viele Menschen zur bevorzugten Geldanlage, auch begünstigt durch die in Deutschland ausgeprägten Vorbehalte gegenüber Aktien und anderen Anlageformen (Just et al., 2017). Durch staatliche Förderung wie z. B. das Baukindergeld wurden zusätzliche Anreize zum Erwerb von Wohneigentum gesetzt und dadurch zur damit einhergehenden Fremdkapitalaufnahme. So ist der Eigenheimerwerb vor allem für Familien und Haushalte mit mittleren Einkommen attraktiver geworden (Voigtländer und Sagner, 2019).

Da die meisten Finanzierungen in Deutschland solide ausgestaltet sind (Hüther et al., 2015), führen kurzzeitige Einkommensrückgänge nicht automatisch zum Verlust des noch nicht vollständig abbezahlten Eigenheims. Darüber hinaus gibt es für Hauseigentümer, die in eine finanzielle Schieflage geraten, auch ohne zusätzliche staatliche Maßnahmen verschiedene Möglichkeiten, sich finanziell zu entlasten, z. B. durch vertraglich mit dem Kreditgeber vereinbarte Tilgungsaussetzungen und Tilgungssatzwechsel oder durch Ratenkredite zur Bedienung der Hypothekendarlehen. Zehn Jahre nach der Auszahlung des Darlehens besteht ohnehin ein Sonderkündigungsrecht ohne Vorfälligkeitsentschädigung, wodurch eine Umschuldung zu im Regelfall deutlich niedrigeren Zinsen möglich wird. Auf all diese Möglichkeiten sollten Betroffene verstärkt aufmerksam gemacht werden.

Erlauben die vertraglichen Bedingungen keinen Ausweg aus der Corona-bedingten Krisenlage, ist der Kreditnehmer auf ein Entgegenkommen seines Kreditgebers angewiesen. Der Umstand, dass auf ein solches Entgegenkommen kein Rechtsanspruch besteht, rechtfertigt die im Gesetz zur Abmilderung der Folgen der COVID-19-Pandemie ermöglichte, begründungspflichtige Stundung der Kreditraten für Verträge, die vor dem 15. März 2020 abgeschlossen wurden. Dies gilt zunächst für Rückzahlungs-, Zins- oder Tilgungsleistungen, die zwischen dem 1. April 2020 und dem 30. Juni 2020 fällig werden. Für diesen Zeitraum entfallen Verzugszinsen und eine Kündigung des Darlehensvertrags aufgrund von Zahlungsverzug oder durch eine verschlechterte Werthaltigkeit von Sicherheiten ist nicht möglich. Das Vertragsverhältnis verlängert sich um die Dauer der Stundung, wodurch vermieden wird, dass mehrere Raten gleichzeitig fällig werden.

### Wohnungsbezogene Sozialpolitik

Hinsichtlich der wohnungsbezogenen Sozialpolitik konzentrieren sich die Sofortmaßnahmen auf die Subjektförderung, was insofern problemadäquat ist, als sich das Leistungsniveau und die -dauer in den einschlägigen Transferleistungssystemen nach den individuellen Unterstützungsbedarfen orientieren. Die subjektorientierten Leistungen sind in zwei Leistungssystemen angesiedelt. Im Rahmen der Grundsicherung nach SGB II (Grundsicherung für Arbeitsuchende) und SGB XII (Grundsicherung im Alter und bei Erwerbsminderung sowie Hilfe zum Lebensunterhalt) werden die Kosten der Unterkunft vollständig übernommen, sofern diese angemessen sind und der Grundsicherungsempfänger mit eigenem Einkommen und Vermögen seinen sonstigen Lebensunterhalt nicht bestreiten kann. Bei überhöhten, also über den lokal ermittelten Angemessenheitsgrenzen liegenden Unterkunftskosten werden diese bisher bis zu sechs Monate in vollem Umfang übernommen, danach aber nur anteilig anerkannt.

Das Wohngeld ist zusammen mit anderen Leistungen wie z.B. dem Kinderzuschlag nach § 6a Bundeskindergeldgesetz gegenüber den Grundsicherungsleistungen vorrangig in Anspruch zu nehmen. Das Wohngeld stellt dabei „nur" einen Zuschuss zu den (kalten) Unterkunftskosten dar, der unter sonst gleichen Bedingungen mit der Haushaltsgröße und mit den Unterkunftskosten – begrenzt durch wohngeldrechtlich vorgegebene, nach Haushaltsgröße und lokaler Mietenstufe ausdifferenzierte Höchstbeträge – steigt und mit dem Haushaltseinkommen sinkt.

Mit dem Sozialschutz-Paket (BGBl, 2020b) hat der Gesetzgeber für Bedarfs- und Einstandsgemeinschaften, deren SGB II- bzw. SGB XII-Leistungsbezug in der Zeit vom 1. März bis 30. Juni 2020 beginnt, Sonderregelungen beschlossen. Zum einen wird auf eine Vermögensprüfung verzichtet und von den Antragstellern lediglich eine Erklärung gefordert, dass kein erhebliches Vermögen vorliegt. Zum anderen werden die Unterkunftskosten für die ersten sechs Monate in voller Höhe übernommen, auch wenn diese über den Angemessenheitsgrenzen liegen. Erst danach folgt das sogenannte Kostensenkungsverfahren, in dem für maximal weitere sechs Monate höhere Unterkunftskosten anerkannt werden – es sei denn, der Grundsicherungsträger sieht in Einzelfällen aus Härtefallgründen oder auf Basis von Wirtschaftlichkeitsüberlegungen dauerhaft von Maßnahmen zur Kostensenkung ab. Durch die Sofortmaßnahmen im Sozialschutz-Paket soll eine unbürokratische und schnelle Unterstützung für die Bevölkerungskreise ermöglicht werden, die Corona-bedingt massive Einkommensverluste erleiden. Gleichzeitig sollen die Grundsicherungsträger bei der Bearbeitung der Leistungsanträge entlastet werden.

Diese Sonderregelungen sind unter der Annahme, dass es sich bei einer relevanten Zahl um Fälle mit temporären Einkommensproblemen handelt, problemadäquat. Das deutsche Grundsicherungssystem ist darauf ausgerichtet, ein Abrutschen von Haushalten unter das soziokulturelle Existenzminimum wirksam zu unterbinden. Durch die vereinfachte Antragsprüfung wird nicht nur das Stigma des Grundsicherungsbezugs reduziert, sondern zugleich das Verwaltungshandeln entlastet, um den Betroffenen zeitnah die entsprechenden Leistungen zu gewähren. Dass von den niederschwelligen Sofortmaßnahmen unter Umständen auch nicht krisenbedingte Fälle profitieren, ist angesichts der mit dem Sozialschutz-Paket intendierten gesetzgeberischen Ziele in Kauf zu nehmen. Allerdings könnten Bedarfs- und Einstandsgemeinschaften, die bereits vor der Pandemie im Leistungsbezug waren und aufgrund zu hoher Unterkunftskosten eine Kostensenkungsaufforderung erhielten, aufgrund des derzeitigen Einbruchs des Vermietungsgeschehens Probleme haben, innerhalb der erwähnten Sechsmonatsfrist ihre Kosten durch einen Umzug zu senken. Folgerichtig ist auch für diese Fälle – gegebenenfalls auf Basis eines standardisierten Nachweises der Anmietungsversuche – eine längere Übernahme höherer Unterkunftskosten geboten. Angesichts der Relevanz der Kostenübernahme für die Bedarfsdeckung und der zugleich sehr uneinheitlichen Praxis der Grundsicherungsträger bei der Prüfung der konkreten Verfügbarkeit von Wohnraum (Malottki et al., 2017) wären für Fälle, bei denen zu Beginn der Kontaktsperren ein Kostensenkungsverfahren anhängig war, eine Übergangsregelung oder zumindest gesetzgeberische Standards der Verfügbarkeitsprüfung dringend zu prüfen.

Zu beachten ist auch, dass die Sofortmaßnahmen im SGB II und XII zulasten der vorrangigen Leistungen gehen, da dort – mit Ausnahme des nur für Familien infrage kommenden Kinderzuschlags – keine Leistungsverbesserungen vorgesehen wurden. Um allein durch die Sofortmaßnahmen induzierte Wechsel vom Wohngeld in die Grundsicherung zu unterbinden und um „Neufällen" einen Eintritt in das autonomieschonendere, anreizkompatiblere und weniger stigmatisierte Wohngeldsystem zu ermöglichen,[1] sollten für den gleichen Bewilligungszeitraum und ebenfalls für sechs Monate parallele Regelungen im Wohngeldsystem eingeführt werden. Konkret sollte die Begrenzung der Unterkunftskosten auf die wohngeldrechtlichen Höchstbeträge genauso ausgesetzt werden wie die wohngeldrechtliche Vermögensprüfung. Von der Aussetzung der Höchstbeträge würden dabei auch aktuelle Wohngeldbezieher mit Unterkunftskosten oberhalb der Höchstbeträge profitieren, die gegenwärtig bis zu einem Drittel aller Wohngeldhaushalte ausmachen dürften.[2] Die Begünstigung solcher „Altfälle" wäre in Kauf zu nehmen, da diese sonst in das durch die Sofortmaßnahmen modifizierte Grundsicherungssystem abrutschen und so die intendierte Arbeitsentlastung der Grundsicherungsträger ein Stück weit unterlaufen könnten.

Gerade für selbstnutzende Eigentümer in den Anfangsjahren einer annuitätenbasierenden Darlehensfinanzierung ist das Wohngeldsystem gegenüber der Grundsicherung bereits heute attraktiv, weil das Wohngeld anders als die

1 Zur Problematik von Wechseln zwischen Grundsicherung und Wohngeld und den Folgen für die Betroffenen vgl. Cischinsky und Krapp, 2020. Zur positiven Anreizstruktur des Wohngelds vgl. Henger und Niehues, 2019.

2 Die Überschreiterquote lag 2016 bei 20 % und 2017 bei 23 % und dürfte seitdem weiter angestiegen sein (BMI, 2019).

Grundsicherung nicht nur Fremdkapitalzinsen, sondern auch Tilgungsleistungen bezuschusst. Von der empfohlenen temporären Aussetzung der wohngeldrechtlichen Höchstbeträge würde dieser Personenkreis daher in besonderer Weise profitieren.

## Grundlegende Charakteristika der Marktentwicklung

Die Unsicherheit über die Dauer der aktuellen Einschränkungen sowie über Erholungspfade der Weltwirtschaft macht eine Prognose der mittel- bis langfristigen Folgen für den deutschen Wohnungsmarkt schwierig. Dennoch lohnt ein Blick auf die Charakteristika der vergangenen Entwicklung des deutschen Wohnungsmarkts, um wesentliche Treiber krisenhafter Entwicklungen zu erkennen. Auf dieser Grundlage werden Szenarien möglicher Auswirkungen auf die Preisentwicklung diskutiert und wohnungspolitische Handlungserfordernisse abgeleitet. Die Darstellung in Bezug auf Mieten beschränkt sich dabei auf die Entwicklung in den sieben größten deutschen Städten, die rund 20 % des Mietwohnungsbestands umfassen und aufgrund ihrer Entwicklungsdynamik am ehesten geeignet sind, Zusammenhänge zu illustrieren. Die grundlegenden Prozesse dürften in der breiteren Marktentwicklung mit entsprechend geringerer Dynamik ablaufen.

### Preisvolatilität

Seit Mitte der 2000er Jahre sind in Deutschland nach einer längeren Phase stagnierender oder rückläufiger Preise deutlich steigende Miet- und Kaufpreise für Wohnimmobilien zu beobachten. In der Betrachtung der sieben größten Städte ist über die letzten fast 50 Jahre erkennbar, dass Marktmieten inflationsbereinigt weitgehend stationär verliefen, d. h. im Mittel auf ein in etwa vergleichbares Niveau zurückkehrten, jedoch unterschiedlich starke zyklische Ausschläge aufwiesen (vgl. Abbildung 1). Die aktuelle Entwicklung steigender Mietpreise begann etwa Mitte der 2000er Jahre und erreicht in realen Größen nicht ganz das Niveau des ausgeprägten Hochpunkts Mitte der 1990er Jahre. Weniger ausgeprägte Hochphasen sind Anfang der 1970er, Anfang der 1980er Jahre und in der ersten Hälfte der 2000er Jahre erkennbar. Der Index der Kaufpreise zeigt ein vergleichbares Bild; die Entwicklung war aufgrund der breiteren geografischen Datenbasis und der geringeren Liquidität im Wohneigentumssegment jedoch weniger volatil.

Als Treiber der Preisentwicklung wird häufig die Niedrigzinspolitik der EZB genannt. Dieser Erklärungsansatz kann zwar die Intensität von zyklischen Preisschocks (Glaeser et al., 2008) erklären, eignet sich jedoch nicht zur Begründung, warum es zur Trendwende auf dem Wohnungsmarkt gekommen war. Zum einen ist erkennbar, dass die Wohnbaukreditzinsen bis zur weltweiten Finanzkrise 2008 prozyklisch mit Mieten und Kaufpreisen korrelierten. Phasen steigender Mieten waren also durch vergleichsweise höhere Kreditzinsen geprägt. Diese Entwicklung lässt das aktuelle antizyklische Zinspreisregime ohne historische Parallelität erscheinen. Zum anderen erklären Zinseffekte Veränderungen im Mietpreisniveau kaum, da die Mietpreisbildung in stark bestandsdominierten Märkten wie dem Wohnimmobilienmarkt kurzfristig nicht von den Kosten der Finanzierung abhängt. Im Lichte der langfristigen Entwicklung lässt sich ein geringeres Zinsniveau als Ursache der zyklischen Mietpreisentwicklung daher weitgehend ausschließen.

Abbildung 1
**Mieten, Kaufpreise und Wohnbaukreditzinsen**

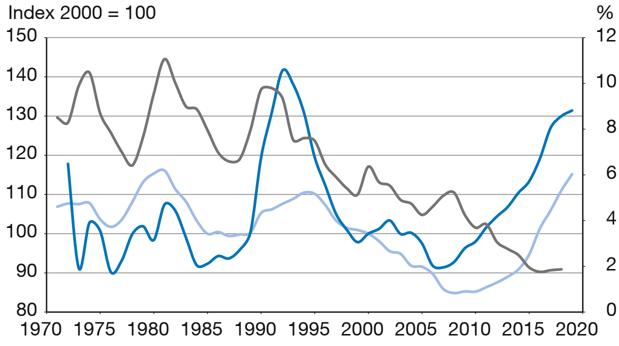

Anmerkungen: *Mieten:* bis 2009 eigene Berechnung auf Grundlage des RDM (ivd)-Preisspiegels, Erstvermietungsmieten mittlerer Wohnwert (bezogen auf ca. 3 Zimmer, 70 qm), Top-7-Städte Deutschland (einwohnergewichtet), ab 2010 auf Grundlage Deutsche Bundesbank, Mieten für Eigentumswohnungen 7 Großstädte (A-Städte, transaktionsbezogene Objekt- und Regionalgewichtung). *Kaufpreise:* Wohnimmobilienpreisindex Deutsche Bundesbank – lange Zeitreihe (Kaufpreise für Wohnimmobilien, wechselnde regionale Zusammensetzung). *Wohnbaukreditzinsen:* Effektivverzinsung, Deutsche Bundesbank, bis Mai 1982 Kredite mit Zinsvereinbarungen aller Art; ab Juni 1982 Hypothekarkredite zu Festzinsen auf fünf Jahre. Ab Januar 2003 Zinssätze für Wohnungsbaukredite an private Haushalte.

### Wohnraumnachfrage: volatile Außenwanderung als Treiber

Der auffällige Gleichlauf von Marktmieten und Außenwanderungsintensität aus dem europäischen Ausland über die letzten fünf Dekaden verweist auf Migration als Treiber der zyklischen Marktmietenentwicklung (vgl. Abbildung 2). Die Preisvolatilität ist damit vorrangig Folge von zeitlich befristeten Nachfrageschocks auf dem Wohnungsmarkt. Die Außenwanderung spielt hier eine

Abbildung 2
**Außenwanderungssaldo und Mieten**

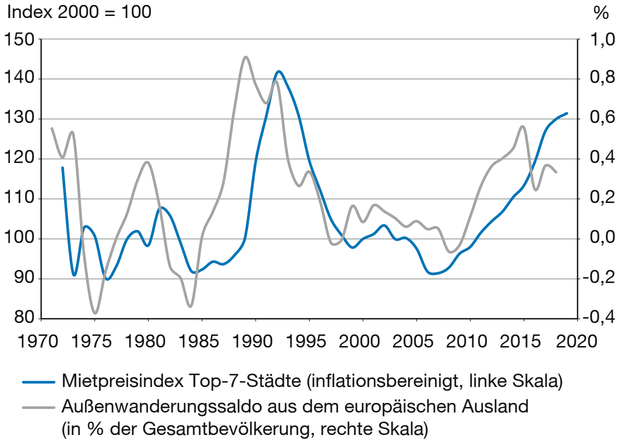

Anmerkungen: *Außenwanderungssaldo:* eigene Berechnung auf Grundlage des Statistischen Bundesamts, Fortschreibung des Bevölkerungsstands, Wanderungsstatistik (bis 1991 Gebietsstand früheres Bundesgebiet). *Mieten:* vgl. Abbildung 1.

Abbildung 3
**Wohnbautätigkeit und Mieten**

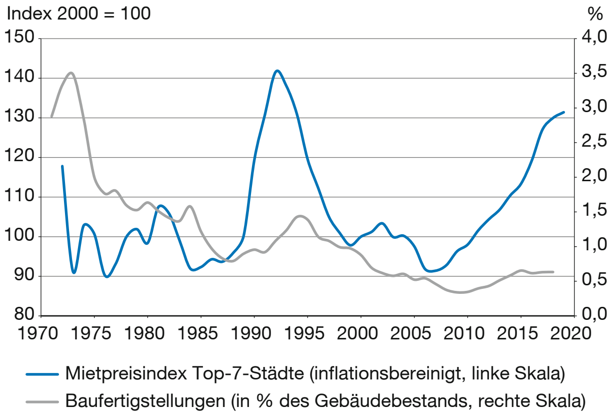

Anmerkungen: *Wohnbautätigkeit:* eigene Berechnung auf Grundlage des Statistischen Bundesamts, Baufertigstellungsstatistik, Fortschreibung des Wohnungsbestandes (bis 1991 Gebietsstand früheres Bundesgebiet). *Mieten:* vgl. Abbildung 1.

herausragende Rolle, denn im Gegensatz zur Binnenwanderung generiert Zuwanderung eine unmittelbare Zusatznachfrage. Auch quantitativ ist sie von zentraler Bedeutung für die Wohnungsnachfrage: Im aktuellen Zyklus umfasst der Außenwanderungssaldo aus dem europäischen Ausland seit 2011 im Jahresmittel (ohne Berücksichtigung des durch den starken Flüchtlingszustrom geprägten Jahres 2015) ca. 280.000 Personen pro Jahr, entsprechend 0,35 % der Bevölkerung.[3] Bei einer mittleren Wohnbautätigkeit von ca. 230.000 Einheiten pro Jahr oder ca. 0,54 % des Wohnbestands entspräche der Anteil der von Außenwanderung ausgelösten Wohnbedarfe, gleiche Haushaltsgrößen wie in der Residenzbevölkerung vorausgesetzt, knapp zwei Drittel der gesamten Wohnbautätigkeit im Vergleichszeitraum, bei kleineren Haushalten sogar noch mehr.

Ungeachtet der Sondersituation der wiedervereinigungsbedingten Zuwanderung nach Deutschland – allein in der Dekade ab 1990 wanderten 2,1 Mio. (Spät-)Aussiedler nach Deutschland zu, davon ca. 81 % aus den Staaten der ehemaligen Sowjetunion (Worbs et al., 2013) – stellt die Arbeitsnachfrage den Haupttreiber der Zuwanderungstätigkeit dar. In den urbanen Arbeitsmärkten korrelieren Binnen- und Außenwanderung positiv miteinander, was die Nachfragesituation in den betroffenen Wohnungsmärkten besonders anspannt. In den sieben größten deutschen Städten betrug der Anteil der Außenwanderung laut amtlicher Statistik seit 2011 im Mittel knapp 50 % der gesamten Zuwanderung. Neben der inländischen Entwicklung auf dem Arbeitsmarkt als Pullfaktor spielt die Erwerbssituation in den Herkunftsländern als Pushfaktor eine zentrale Rolle. Die Zuwanderung im aktuellen Zyklus wurde dabei besonders stark durch die verzögerte wirtschaftliche Erholung wichtiger Herkunftsländer im Zuge der Eurokrise bestimmt. Während im zeitlichen Zusammenhang mit der Finanzkrise 2008 und 2009 leichte Außenwanderungsverluste verzeichnet wurden, nahmen die Wanderungsgewinne ab 2011 besonders stark zu.

## Bautätigkeit: unelastisches Angebot erhöht Preisvolatilität

Die langfristige Entwicklung der Neubautätigkeit in Deutschland zeigt einen negativen Trend (vgl. Abbildung 3). Lagen Anfang der 1970er Jahre die jährlichen Fertigstellungsraten noch bei über 3 % des Gesamtbestands,[4] gingen sie bis 2009 auf unter 0,4 % zurück. Dieser Trend wurde regelmäßig von Phasen zyklisch gesteigerter Neubautätigkeit unterbrochen. Diese folgen den jeweiligen Nachfrageschocks und zeigen ein relativ träges Reaktionsmuster. Die Beobachtungen bestätigen die strukturell vergleichsweise geringe Preiselastizität der

3 Dem steht ein seit 1972 durchwegs negativer natürlicher Bevölkerungssaldo (definiert als die Zahl der Neugeborenen abzüglich der Zahl der Gestorbenen) gegenüber, seit 2011 im Mittel um ca. -180.000 Personen pro Jahr.

4 Hohe Fertigstellungsintensitäten waren vor allem zum Abbau der kriegsbedingten Wohnraumunterversorgung erforderlich.

Wohnbaufertigstellungen in Deutschland (Caldera und Johansson, 2013), die Preise in Phasen zunehmender Nachfrage schneller steigen lässt. Der Zeitversatz zwischen Nachfrage und angebotsseitiger Reaktion erhöht in Phasen rückläufiger Nachfrage zudem die Gefahr von Angebotsüberhängen, die zu einem beschleunigten Rückgang der Marktpreise führen. Beide Effekte sind maßgeblich für die beobachtbare Preisvolatilität verantwortlich.

## Szenarien mittel- bis langfristige Marktentwicklungen

Die weitere Entwicklung des deutschen Wohnungsmarkts ist weitgehend von der zukünftigen wirtschaftlichen Entwicklung abhängig und kann wegen der erheblichen Unsicherheit über Stärke und Länge der erwarteten Rezession im Folgenden nur skizziert werden. Dabei wird zwischen einer mittelfristigen rezessiven Phase und einer längerfristigen Erholungsphase unterschieden.

### Mittelfristige Entwicklungen

Die Wohninfrastruktur in Deutschland hat durch die Entwicklung in der letzte Dekade einen sehr hohen Auslastungsgrad mit zum Teil erheblicher Unterversorgung – gemessen an der Wohnversorgungsquote – in den größeren Städten erreicht (Vaché, 2016). Jedoch zeichnet sich seit 2016 ein rückläufiger Trend der Außenwanderungsgewinne aus dem europäischen Umfeld ab, verursacht durch eine allmähliche Verbesserung der Arbeitsmarktsituation in den Herkunftsländern. Vor dem Hintergrund der skizzierten langfristigen Strukturmerkmale des deutschen Wohnungsmarkts ist dies als Frühindikator für eine Absenkung des Mietpreisniveaus in Deutschland zu werten. Bei Corona-bedingt beschränkter Freizügigkeit ist mittelfristig von einer Phase mit geringerer Zuwanderungsintensität auszugehen, was angesichts der Erfahrungen der Vergangenheit mittelfristig zu einem Rückgang der besonders volatilen realen Erstvermietungspreise in Großstädten in einer Größenordnung von ca. 30 % bis 40 % gegenüber dem Spitzenniveau der vergangenen Jahre führen kann (vgl. Abbildung 2).[5]

Angesichts der aktuell bestehenden Wohnungsdefizite resultiert aus diesem Szenario zunächst eine Entspannung in den Ballungsräumen. Dazu könnte auch ein möglicher Rückgang der touristischen und gewerblichen Nachfragekonkurrenz nach Wohnraum in den Großstädten beitragen, was den Anreiz zu einer Rückkehr in das reguläre Wohnungsmietgeschäft erhöhen würde. Eine Zunahme des Wohnungsangebots bei rückläufigen Mietpreisen wird nach der Lockerung der Corona-bedingten Kontaktverbote zu einer Belebung der bereits zuvor reduzierten innergemeindlichen Umzugstätigkeit führen. Vor allem ortsansässige Haushalte werden ihre verbesserte Wettbewerbsposition zur Nachholung bislang unterlassener Anpassungen des Wohnkonsums nutzen und damit zu einer insgesamt besseren Auslastung der Wohninfrastruktur beitragen. Eine gesteigerte Transaktionstätigkeit auf den Miet- und Eigentumswohnungsmärkten dürfte daher die negative Preisentwicklungsdynamik dämpfen.

Angebotsseitig sind wegen der langen Produktionszyklen im Wohnungsbau die Möglichkeiten zur kurzfristigen Anpassung der Fertigstellungszahlen beschränkt. Dazu tragen auch die zuletzt sehr hohen Baulandpreise bei, die die Kosten einer Verzögerung der Vermarktungsphase für die auf den betreffenden Grundstücken gebauten Immobilien erhöhen. Es steht daher zu erwarten, dass die gestiegenen Wohnbaufertigstellungszahlen 2020 und gegebenenfalls auch noch 2021 aufrechterhalten werden und zur beschriebenen Entspannungstendenz beitragen. Kommt es infolge anhaltender Störungen im grenzüberschreitenden Baumaterial- und Arbeitskräfteverkehr, etwa durch wiederholte Grenzschließungen, dagegen zu Kapazitätsengpässen, können die Baufertigstellungszahlen auch schneller sinken.

### Langfristige Entwicklungen

Die weitere Entwicklung der Nachfragesituation auf dem deutschen Wohnungsmarkt wird erwartungsgemäß von der relativen wirtschaftlichen Dynamik in Deutschland und den traditionellen Herkunftsländern der Arbeitsmigration abhängen. Eine schnellere wirtschaftliche Erholung in Deutschland dürfte die ausländische Nachfrage langfristig wieder ansteigen lassen. Inwieweit die in der jüngeren Vergangenheit für die deutsche Wohnungsnachfrage prägenden asynchronen Arbeitsmarktzyklen zwischen Herkunfts- und Zielländern der Arbeitsmigration auch den wirtschaftlichen Erholungspfad aus der aktuellen Krise prägen werden, ist allerdings noch nicht abzusehen.

Auf der Angebotsseite ist bei anhaltender Unsicherheit über die Nachfrageentwicklung eine Verschiebung oder Stornierung von Bauvorhaben zu erwarten, sodass der Bauüberhang, d. h. die Zahl genehmigter, aber noch nicht fertiggestellter Wohnungen, in den kommenden Jahren weiter zunehmen dürfte. Bei einem stärkeren Rückgang der Neubautätigkeit infolge einer längerfristigen Störung des gesamtwirtschaftlichen Gleichgewichts erhöht sich insgesamt die Gefahr, die Kapazitäten der Bauwirtschaft nachhaltig zu schwächen und daher bei einer Erholung

5 Die Preisvolatilität in anderen Marktsegmenten (z. B. bei Bestandswohnungen oder im Neubausektor außerhalb von Großstädten) fiel in der Vergangenheit geringer aus, wovon auch hier ausgegangen wird.

der arbeitsmarktbedingten Auslandsnachfrage in eine erneute Angebotslücke am Wohnungsmarkt zu laufen.

### Mittel- bis langfristige wohnungspolitische Handlungserfordernisse

Der zu erwartende mittelfristige Preisrückgang auch kreditfinanzierter Wohnimmobilien dürfte die Stabilität des Finanzwesens nicht gefährden, da anzunehmen ist, dass die nachhaltig erzielbaren Mieten und Kaufpreise bei der Ermittlung von Beleihungswerten regulierungsbedingt adäquat berücksichtigt wurden und darüber hinausgehende Risiken durch entsprechende Sicherheiten abgedeckt sind. Aufgrund der geringen Bedeutung von Immobilien als potenziell liquide Vermögensposition und der traditionell langen Haltedauern sind auch keine schwerwiegenden Folgen sinkender Preisniveaus für das Konsum- oder Finanzierungsverhalten der Haushalte zu erwarten (Heijden et al., 2011). Die trotz Intensivierung noch unter dem Bedarfsniveau liegende Neubautätigkeit der letzten Jahre (Held und Waltersbacher, 2015) reduziert auch die Gefahr eines spekulativen Überangebots, das im Zuge der Finanzkrise 2008 zur Destabilisierung des Immobilienfinanzierungssektors geführt hat. Zur Stabilisierung des Finanzsektors erscheinen Stützungsinstrumente auf Haushaltsebene daher aktuell nicht geboten.

Gleichwohl ist nicht auszuschließen, dass aufgrund rezessionsbedingter Einkommensrückgänge ein Teil der kreditfinanzierenden Eigenheimbesitzer zunehmend vor schwerwiegenden Finanzierungsproblemen steht. Gerade weil durch die – aufgrund ihrer problematischen Anreiz- und Verteilungswirkungen umstrittene – eigentumsorientierte Förderpolitik zusätzliche Anreize zum Erwerb von Wohneigentum gesetzt wurden, erscheint eine wohnungspolitische Reaktion angemessen, um drastische Folgen für die betroffenen Haushalte abzuwenden. Denkbar wäre eine gezielte Stärkung des Wohngelds durch ein längerfristiges Aussetzen der wohngeldrechtlichen Höchstbeträge bei betroffenen Eigenheimbesitzern.

Die 2015 eingeführte und 2020 verlängerte Mietpreisbremse wird auch im mittelfristigen Szenario fallender Marktmieten als unkritisch gesehen, auch wenn dies ihrer Steuerungsintention entgegensteht. Trotzdem erscheint vor dem Hintergrund potenziell wiederkehrender Probleme mit volatilen Marktbedingungen eine Verstetigung mietpreisdämpfender Regulierungsinstrumente diskussionswürdig. So dürfte die jüngst eingeführte Verlängerung des Bemessungszeitraums bei der Ermittlung von ortsüblichen Vergleichsmieten von vier auf sechs Jahre zu einer artifiziellen Verlängerung des Hochpreiszyklus in Vergleichsmietpreisinformationen wie Mietspiegeln führen. Bei gleichzeitig fallenden Mietpreisen kann sich die Legitimationsproblematik des Vergleichsmietensystems damit noch verstärken (Arbeitsgemeinschaft der Mietspiegelersteller, 2019).

Grundsicherung wie auch Wohngeld sind bewährte nachfrageseitige Maßnahmen, um ein angemessenes Wohnen auch in Zeiten wegbrechender Einkommen zu ermöglichen. Staatliche Maßnahmen sind im Fall einer Rezession daher grundsätzlich nicht geboten. Neben der vorgeschlagenen wohngeldrechtlichen Härtefallregelung bei kreditfinanzierenden Eigenheimbesitzern wäre in der Grundsicherung angesichts potenziell sinkender Angemessenheitsgrenzen eine sozialrechtliche Bestandsschutzklausel zu prüfen, um zu verhindern, dass vormals angemessen wohnende Grundsicherungsempfänger plötzlich zur Kostensenkung aufgefordert werden.

Bei einer wachsenden Nachfrage im niedrigpreisigen Segment und einem sich bereits vor der Corona-Krise verstärkenden politischen Anspruch, auch die „breiten Schichten der Bevölkerung" mit gefördertem Wohnraum zu versorgen, wird der Druck auf die geförderten Bestände und damit auf die soziale Wohnraumförderung weiter wachsen. Zugleich ist mit einer rezessionsbedingten Zunahme von Dringlichkeitsfällen zu rechnen. Auch konjunkturpolitisch erscheint eine Stabilisierung bzw. Ausweitung der objektorientierten Förderaktivitäten sinnvoll.

Zur Vermeidung erneuter Kapazitätsengpässe bei der Baulandgewinnung im Fall einer wirtschaftlichen Erholung sollten mittel- und langfristige Kapazitätsplanungen für die Wohninfrastruktur weitgehend aufrechterhalten werden. Dies gilt angesichts der erheblichen Vorlaufzeit vor allem für längerfristige Vorhaben der Baulanderschließung, die nicht wegen aktuell rückläufiger Wohnungsnachfrage revidiert werden sollten (Vaché, 2020).

Die Erfahrungen aus der Finanzkrise in betroffenen Ländern Europas (Scanlon und Elsinga, 2014) zeigen, dass die Aufrechterhaltung der wirtschaftlichen Stabilität des Bausektors zur Sicherstellung der zukünftig erforderlichen Baufertigstellungszahlen im Blick behalten werden sollte (Valence und Runeson, 2011). Der Erhalt grundlegender Kapazitäten der deutschen Bauwirtschaft sollte daher bei staatlichen Konjunkturprogrammen mitgedacht werden.

**Title:** ***Coronavirus Crisis and Housing Policy Challenges***

**JEL Classification:** *R21, 31, H55*
